# Supplementary material for: A multi-stable deployable quadrifilar helix antenna with radiation reconfigurability for disaster-prone areas
Source: Nat Commun. 2023 Dec 21;14:8511. doi: 10.1038/s41467-023-44189-9 (PMC10739743; doi:10.1038/s41467-023-44189-9)
Supplement: Supplementary file 1 — Supplementary Information [file 41467_2023_44189_MOESM1_ESM.pdf]

Supplementary Materials for

A Multi-Stable Reconfigurable Deployable Quadrifilar Helix Antenna with a Sliding Feed for Diaster-Prone Areas

Rosette Maria Bichara[1], Joseph Costantine[1], Youssef Tawk[1], Maria Sakovsky[2]

Affiliations:

[1]Department of Electrical and Computer Engineering, Maroun Semaan Faculty of Engineering and Architecture, American University of Beirut, Beirut 1107 2020, Lebanon.

[2]Department of Aeronautics and Astronautics, Stanford University, Stanford, CA94305, USA.

Table of Contents:

Supplementary Note 1. Directional Couplers 3
Supplementary Note 2. Rate Race Couplers 3
Supplementary Note 3. Effect of Slotted Ground 3
Supplementary Table 1. Antenna performance metrics as a function of helix geometry 4
Supplementary Table 2: Comparison between conventional and proposed QHA 5
Supplementary Table 3: Material properties of a single ply of cured FRP material. 6
Supplementary Figure 1. Power division and combining. (a) Power division. (b) Power combining 6
Supplementary Figure 3. A ring hybrid, or rat-race, in microstrip line or stripline form. 7
Supplementary Figure 5. Total length of each wire of the conventional QHA at the frequency of 1 GHz for axial mode and normal mode in addition to our proposed structure 8
Supplementary Figure 6. Tailoring axial stiffness of two stable states, $K1$ and $K2$, using the fiber angle, θ, and dielectric strip width, w. 8
Supplementary Figure 7: Deployment demonstration using shape memory alloy springs. 9
References 9

Supplementary Notes

Supplementary Note 1. Directional Couplers

Directional[1] couplers are passive microwave components. They can be used for power division or power combining as shown in supplementary Figure 1. The power combiner combines two or more input signals. The power division divides an input signal into two or more output signals. T-junctions and other power dividers are examples of three-port networks. Directional couplers and hybrid couplers are examples of four-port networks as shown in Supplementary Figure 2. Power dividers typically provide in-phase output signals with an equal power division ratio (3 dB). While hybrid junctions typically feature equal power division, directional couplers can be constructed for any type of power division. Hybrid junctions have an output port phase shift of either 90° or 180°. Quadrature hybrid couplers are 3-dB directional couplers. Between the outputs of the through and coupled arms, there is a 90° phase difference. A planar structure is typically designed using microstrip lines.

The 180° hybrid junction is a four-port network. It has a 180° phase shift between its two output ports. An input signal applied to port 1 will be equally divided into two in-phase components at ports 2 and 3. Port 4 will be isolated.

Supplementary Note 2. Rate Race Couplers

The rat-race coupler is shown in supplementary Figure 3. The conventional rat-race coupler is formed of three segments of λ/4 transmission lines and one segment of length 3λ/4. It is characterized by its simple design and its high degree of isolation between the input ports[2]. The rat-race hybrid[1] coupler has a 180° phase difference between ports 2 and 3 when fed at port 4.

Supplementary Note 3. Effect of Slotted Ground

We studied the effect of the added covered slots on the performance of the antenna. We introduced slots into the ground plane of the antenna, which were then covered in order to ensure ground continuity. A comparison between the full ground and the covered slotted ground is shown in Supplementary Figure 4. As can be seen, there is no effect of the slots when covered on the antenna performance. The covered slotted

ground plane acts as a full ground plane so the radiation pattern is very similar when using a full ground plane or a slotted ground plane with covers.

Supplementary Tables

Supplementary Table 1. Antenna performance metrics as a function of helix geometry

**Radius pec (mm)**	**Radius dielectric (mm)**	**S-parameter**	**Radiation pattern**	**AR**	**Frequency (GHz)**	
60	60.6	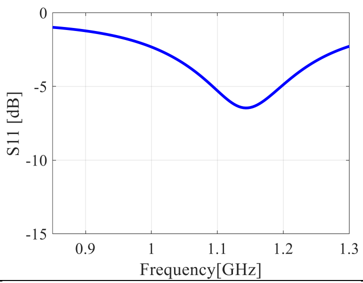	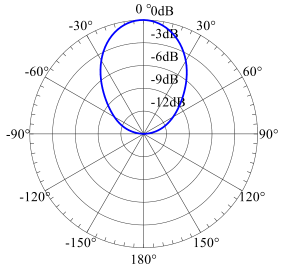	CP	1.143	No matching
55	55.6	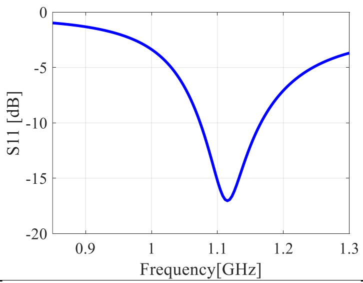	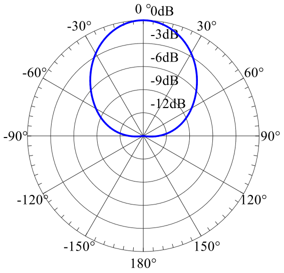	CP	1.115	Matching/ Directional radiation pattern
40	40.6	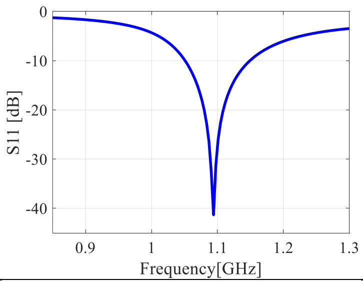	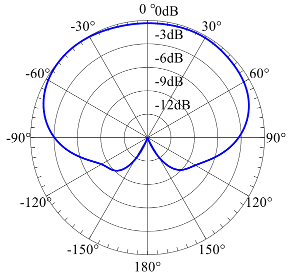	CP	1.094	Matching/ Transition state
35	35.6	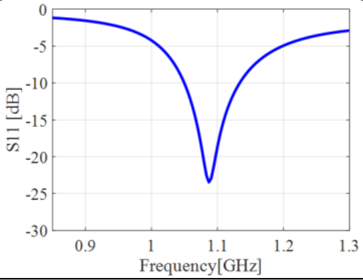	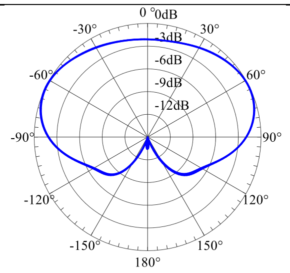	NCP	1.087	Matching/ Transition state

30	30.6	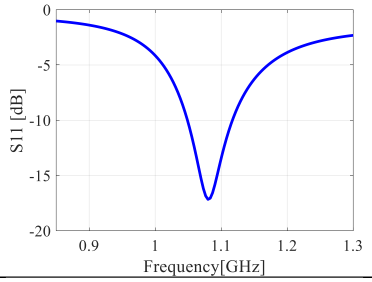	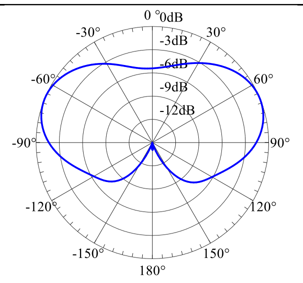	NCP	1.08	Matching/ Omni-directional radiation pattern
25	25.6	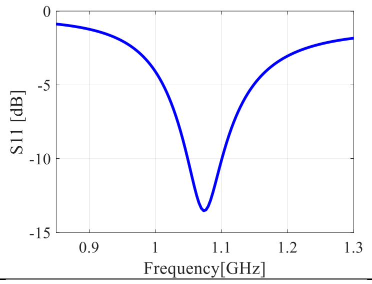	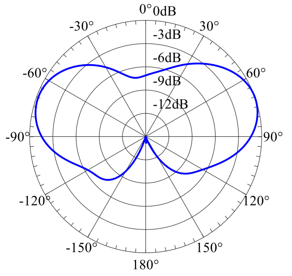	NCP	1.073	Matching/ Omni-directional radiation pattern
20	20.6	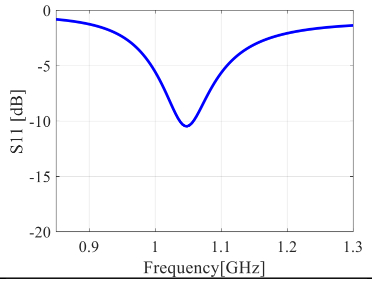	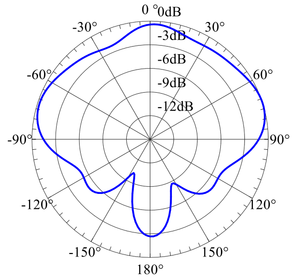	NCP	1.0485	No matching/ Negative gain
10	10.6	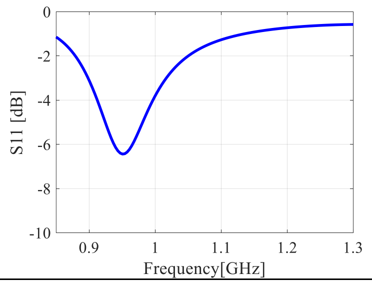	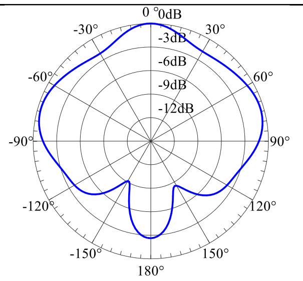	NCP	0.9505	No matching/ Negative gain

*NCP: Not circularly polarized *CP: Circularly polarized

Supplementary Table 2: Comparison between conventional and proposed QHA

**Antenna type**	**Conventional**		**Proposed**	
**Mode**	**Normal**	**Axial**	**Normal**	**Axial**
**Radius, R [mm]**	R$\leq$ 21.7	$32.6 < R < 58.0$	R=30	R=55
**Spacing, S [mm]**	$S \leq 136.0$	$S = 68.3$	$S = 326.1$	$S = 149.5$

Supplementary Table 3: Material properties of a single ply of cured FRP material.

Modulus in Fiber Direction $E_1[GPa]$	Transverse Modulus $E_2[GPa]$	Shear Modulus $G_{12}[GPa]$	Poisson's Ratio ν_{12}	Fiber Volume Content V_f	Areal Weight $[g/m^2]$
41.0	11.5	3.4	0.27	0.55	25

Supplementary Figures:

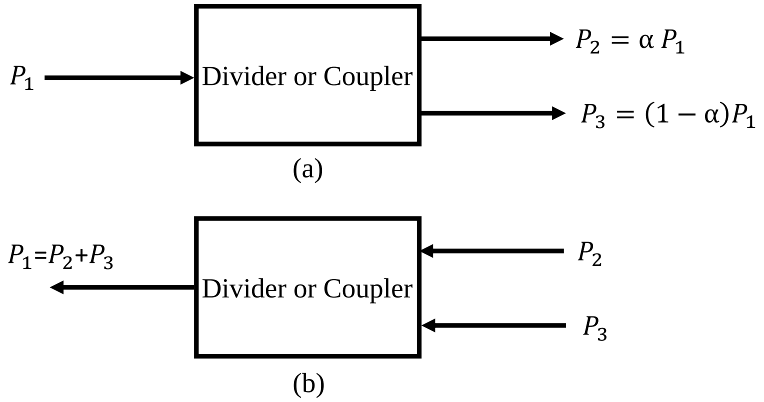

Supplementary Figure 1. Power division and combining. (a) Power division. (b) Power combining

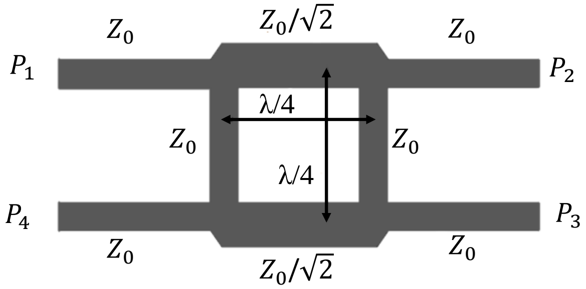

Supplementary Figure 2. The geometry of a branch-line coupler[1]

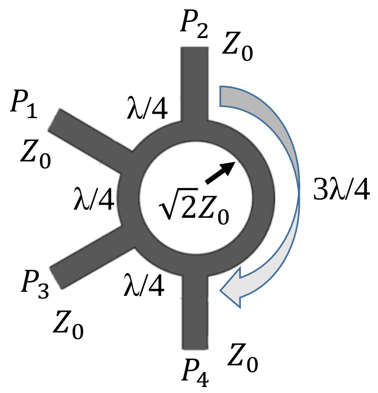

Supplementary Figure 3. A ring hybrid, or rat-race, in microstrip line or stripline form.

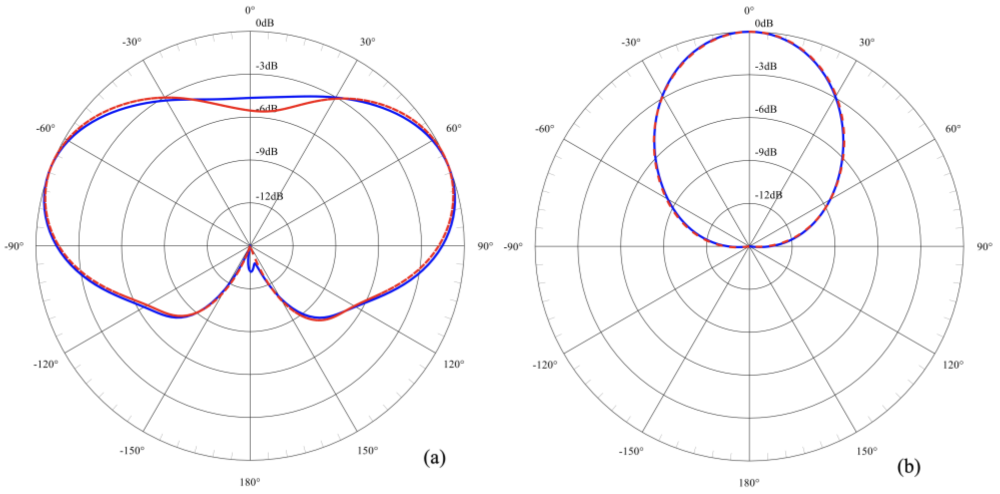

Supplementary Figure 4. Effect of full ground and covered slotted ground plane at 1.1 GHz on (a) radiation pattern for state 1, (b) state 2.

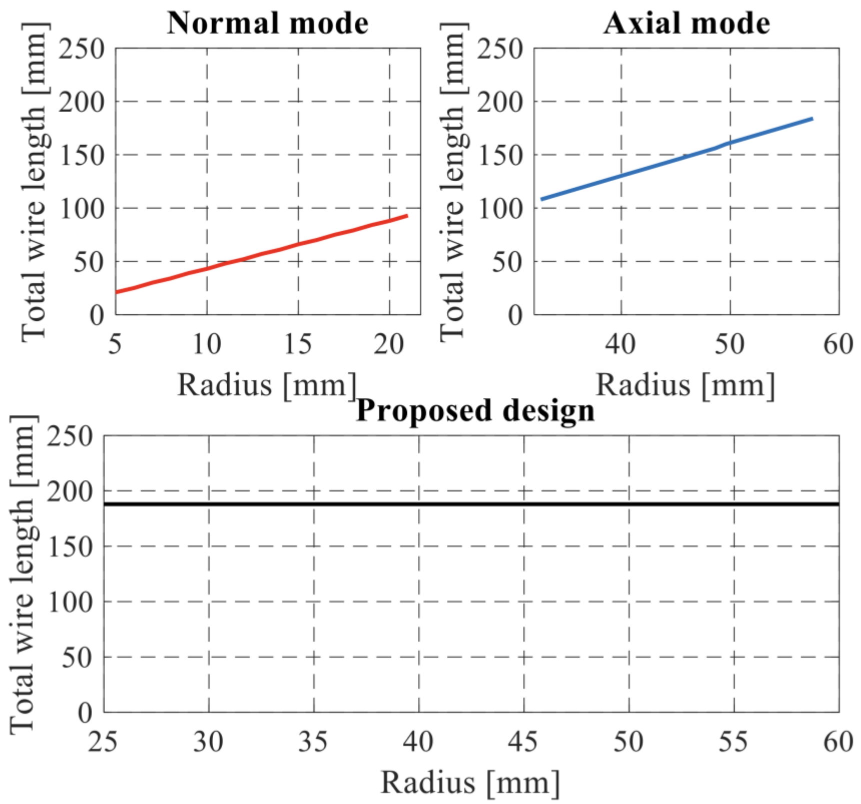

Supplementary Figure 5. Total length of each wire of the conventional QHA at the frequency of 1 GHz for axial mode and normal mode in addition to our proposed structure

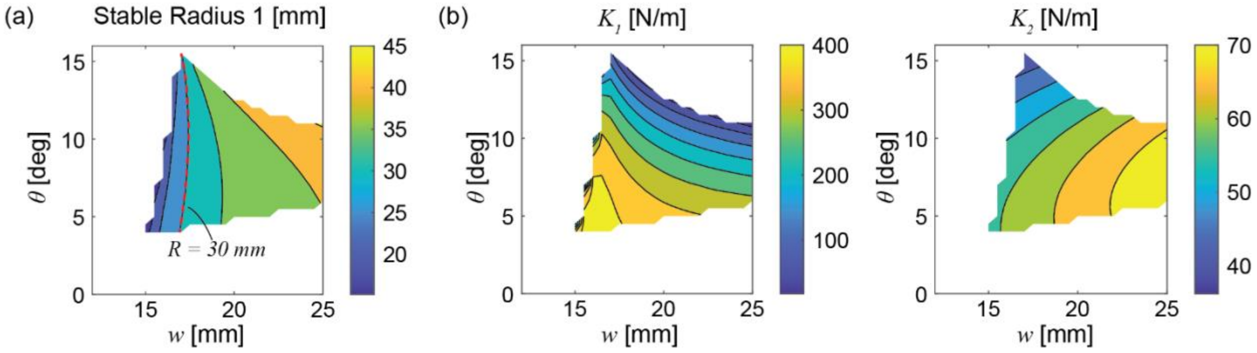

Supplementary Figure 6. Tailoring axial stiffness of two stable states, K_1 and K_2, using the fiber angle, θ, and dielectric strip width, w. The following are used for the analysis: fixed conductive strip width of 8 mm, conductive strip layup of $[\theta_7^{GFRP}/0_{pw}^{PB}/\theta_7^{GFRP}]$, and dielectrip strip layup of $[-\theta_4^{GFRP}/90_3^{GFRP}]_s$. (a) Stable radius in state 1. Note that the stable radius of state 2 is always at the maximum possible radius. (b) Resulting axial stiffness in two stable states. The axial stiffness can be tailored to control the structural resonance in the two states under the constraint of a desired stable radius (e.g., R = 30 mm as in the red line in (a)).

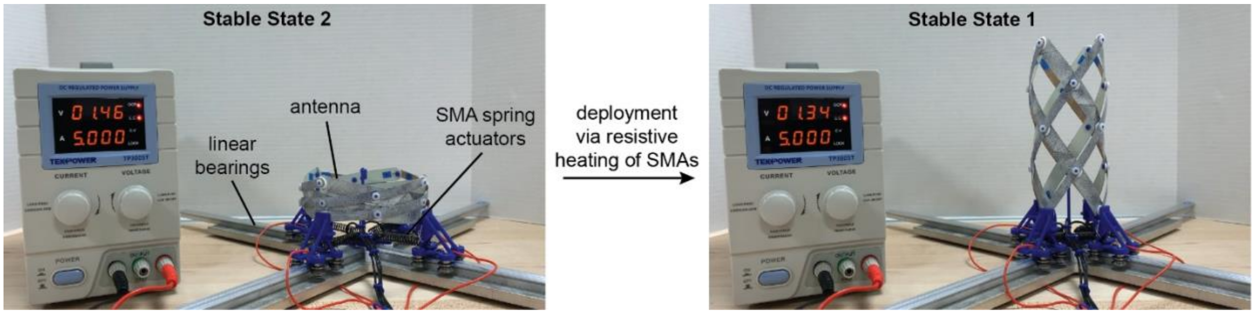

Supplementary Figure 7: Deployment demonstration using shape memory alloy springs. Shape memory alloy springs are stretched to the desired actuation stroke and resistive heating is used to heat up springs allowing them to recover the stroke (shape memory effect) and deploy the antenna. The springs are made of NiTi with an austenite finish temperature of 45°C, a wire diameter of 1 mm, and a spring diameter of 4.75 mm. A fixed actuation current of 5A is used. The unstretched and stretched lengths of the actuators correspond to stable states 1 and 2, respectively.

References

1. D. M. Pozar, Microwave engineering; 3rd ed. Hoboken, NJ: Wiley, 2005. [Online]. Available: https://cds.cern.ch/record/882338 10
2. Xu, H., Wang, G., & Lu, K. (2011). Microstrip rat-race couplers. *IEEE Microwave Magazine*, *12*(4), 117–129.
