# Supplementary material for: A multi-stable deployable quadrifilar helix antenna with radiation reconfigurability for disaster-prone areas
Source: Nat Commun. 2023 Dec 21;14:8511. doi: 10.1038/s41467-023-44189-9 (PMC10739743; doi:10.1038/s41467-023-44189-9)
Supplement: Supplementary file 3 — Description of Additional Supplementary Files [file 41467_2023_44189_MOESM3_ESM.pdf]

<u>Description of additional supplementary files</u>

Supplementary Movie 1: Physical demonstration of antenna multi-stability

.
